# Evaluation of Comparative Efficacy of Levothyroxine Versus Kshar Basti and Kanchanar Guggul in the Treatment of Hypothyroidism: Protocol for a Randomized Controlled Trial

**DOI:** 10.2196/57287

**Published:** 2024-09-10

**Authors:** Satyajit Pandurang Kulkarni, Shweta Parwe

**Affiliations:** 1 Mahatma Gandhi Ayurved College Hospital and Research Centre Datta Meghe Institute of Higher Education and Research Wardha, Maharashtra India

**Keywords:** ayurveda, complementary and alternative medicine, hypothyroidism, levothyroxine, rectal enema, massage therapy, sudation

## Abstract

**Background:**

The thyroid gland is an endocrine gland that has an impact on the body’s general metabolism. Thus, the secretions of the thyroid gland can modify the overall metabolism of the entire body. The prevalence of hypothyroidism is increasing quickly, with rates of 2%-5% in affluent countries and 11% in India. Individuals diagnosed with hypothyroidism need to take medication for the rest of their lives, resulting in significant stress. Therefore, conducting a study in this area is imperative.

**Objective:**

This study aims to assess the effectiveness of the therapeutic enema (Kshar Basti) and oral Kanchanar Guggul in the treatment of hypothyroidism.

**Methods:**

The trial group (n=45) will receive a therapeutic enema (Kshar Basti) followed by oral Ayurvedic drugs for 180 days. The control group (n=45) will be given levothyroxine tablets at a dosage of 1.6 µg/kg/day for the same duration. The objective is to examine the alterations in thyroid stimulating hormone (TSH) levels before and after the treatment.

**Results:**

Any deviation of the serum TSH by more than 20% from the initial values, while keeping triiodothyronine (T_3_), and thyroxine (T_4_) levels within the normal range, will be deemed statistically significant. Consequently, we anticipate a statistically significant variation in serum TSH levels between the therapeutic enema and Kanchanar Guggul treatments. Presently, the drug preparation operations are in progress. We expect to start enrolling patients in June 2024, do data analysis in December 2025, and acquire results by early 2026, marking the end of this trial.

**Conclusions:**

This study will evaluate the efficacy of the therapeutic enema, specifically Kshar Basti, in treating hypothyroidism. Furthermore, more research can determine the efficacy of a therapeutic enema (Kshar Basti) in treating overt hypothyroidism and hypothyroidism during pregnancy.

**Trial Registration:**

Clinical Trials Registry India CTRI/2023/05/052389; https://ctri.nic.in/Clinicaltrials/pmaindet2.php?EncHid=Nzk1NjY=&Enc=&userName=052389

**International Registered Report Identifier (IRRID):**

PRR1-10.2196/57287

## Introduction

### Overview

Thyroid hormones have various impacts on different biological systems. They play a crucial role in regulating many physiological processes, such as the vascular system, heart function, brain function (including cognition and mood), skeletal muscle, and bone health. Hypothyroidism refers to the inadequate production of thyroid gland secretions.

Hypothyroidism is primarily characterized by subclinical hypothyroidism (SCH), where levels of thyroid stimulating hormone (TSH) are elevated, but both free thyroxine (T_4_) and triiodothyronine (T_3_) levels remain within the normal range [1]. Overt hypothyroidism (OH) is the second kind of hypothyroidism, characterized by an increase in TSH, a drop in T_4_ and T_3_ levels, and the presence of clinical symptoms indicating hypothyroidism.

As per recommendations from endocrine societies, SCH is frequently managed with levothyroxine (L-thyroxine). This approach perhaps led to L-thyroxine becoming the most often prescribed medication in the United States starting in 2014, with over 15% of Americans aged 61 years and older using it. A recent extensive study conducted on older adults, known as the TRUST (Thyroid Hormone Replacement for Untreated Older Adults with Subclinical Hypothyroidism Trial), along with a subsequent thorough analysis of randomized controlled trials (RCTs), found no evidence of any advantages of L-thyroxine in terms of symptom relief or improvement in quality of life for patients with SCH [2]. Nevertheless, L-thyroxine is typically recommended indefinitely for SCH, which can be stressful for patients [3].

SCH is a burgeoning lifestyle-related ailment in India. In prosperous countries, the prevalence rate of hypothyroidism ranges from 2% to 5%. However, in India, the prevalence rate is 11% [4].

The prevalence rate of hypothyroidism increases with advanced age [5]. The American Thyroid Association guidelines advise the use of L-thyroxine in individuals with SCH and a TSH level of 10 mIU/L or higher. The supposition of SCH leading to OH is the basis of this statement [6].

There are 2 primary issues associated with L-thyroxine therapy, one of which is the uncertainty around the role of L-thyroxine in SCH. There are conflicting observations regarding it, and the patient must take L-thyroxine for the rest of their lives, which is distressing. Hence, there is an urgent requirement for Ayurvedic therapies in SCH.

Ayurveda is practiced in India, Nepal, and Sri Lanka, and is increasingly being adopted in Western countries. Modern medicine has demonstrated efficacy in managing acute medical emergencies, while Ayurveda may effectively handle chronic conditions that pose challenges for Western medical treatment. Individuals diagnosed with hypothyroidism frequently pursue Ayurvedic remedies [7].

Ayurveda recognizes a limited number of disorders that might be associated with hypothyroidism, and by applying Ayurvedic principles, it is possible to effectively manage this illness. Previous research [8] has examined the effectiveness of Ayurvedic treatment for hypothyroidism in multiple studies [9-11]. Nevertheless, these studies are subject to notable constraints, such as the lack of randomization, the absence of consistent therapy in the control group, a poor sample size, and insufficient follow-up time. We have chosen the following research to revise our protocol to overcome their limitations.

A recent study [10] demonstrated that administering Basti for a duration of 30 days, together with oral intake of 1 gram of Kanchanar Guggul twice a day with tepid water, and Vardhaman Pippali for 12 weeks, yielded better results compared to using only Kanchanar Guggul and Vardhaman Pippali in the same dosage for 12 weeks. However, the study conducted for only 12 weeks is inadequate to induce alterations in TSH concentration. Thus, we selected Kanchanar Guggul as the designated medication.

A study [12] indicated that a therapeutic decoction enema (Kshar Basti) was effective in treating hypothyroidism. A 10-day therapy regimen consisting of oral medications and therapeutic enemas using a decoction called Kshar Basti resulted in significant improvement in the symptoms of hypothyroidism. After 2 months of follow-up, the level of serum TSH decreased to 5.76 mIU/L, while T_3_ and T_4_ remained within the normal range. However, it is important to note that this study is limited to a single example.

### Objectives

#### Research Question

Can Ayurvedic treatments involving Kshar Basti (a therapeutic enema with herbal decoction) and oral administration of Kanchanar Guggul tablet effectively treat SCH?

#### Hypothesis

In SCH, the administration of Ayurvedic interventions, specifically Kshar Basti (decoction enema) followed by oral Kanchanar Guggul, has the potential to decrease elevated TSH levels while maintaining normal T_3_ and T_4_ levels.

#### Primary Objective

The main goal is to ascertain whether Ayurvedic therapy involves the administration of Kshar Basti, which is an herbal decoction enema, followed by an oral tablet. Kanchanar Guggul is efficacious in treating SCH.

#### Secondary Objective

Our secondary goal is to evaluate the appropriateness of Basti Karma (rectal enema therapy) and determine the dropout rate of the Basti course.

## Methods

### Overview

To assess and compare the effectiveness of the therapeutic decoction enema (Kshar Basti) and Kanchanar Guggul with L-thyroxine in the treatment of hypothyroidism, we will administer the therapeutic enema (Kshar Basti) for 24 days, followed by oral intake of Kanchanar Guggul for 153 days (a total of 180 days) in the study group. In the control group, L-thyroxine tablets will be taken for 180 days. The results will then be evaluated.

### Ethical Considerations

This study plan was presented to the Institutional Ethical Committee (IEC) of Mahatma Gandhi Ayurved College, Hospital, and Research Centre, and approval from the IEC has been obtained (Ref. MGACHRC/IEC/May-2022/479, dated May 23, 2022).

The participants of SCH will be informed regarding the ayurvedic procedures and the medications, and their written consent will be obtained on an informed consent form that is prepared in English and Marathi.

Periodically, participants will be required to provide their consent for panchakarma treatments and the collection of blood samples.

### Study Setting

**Figure 1:**
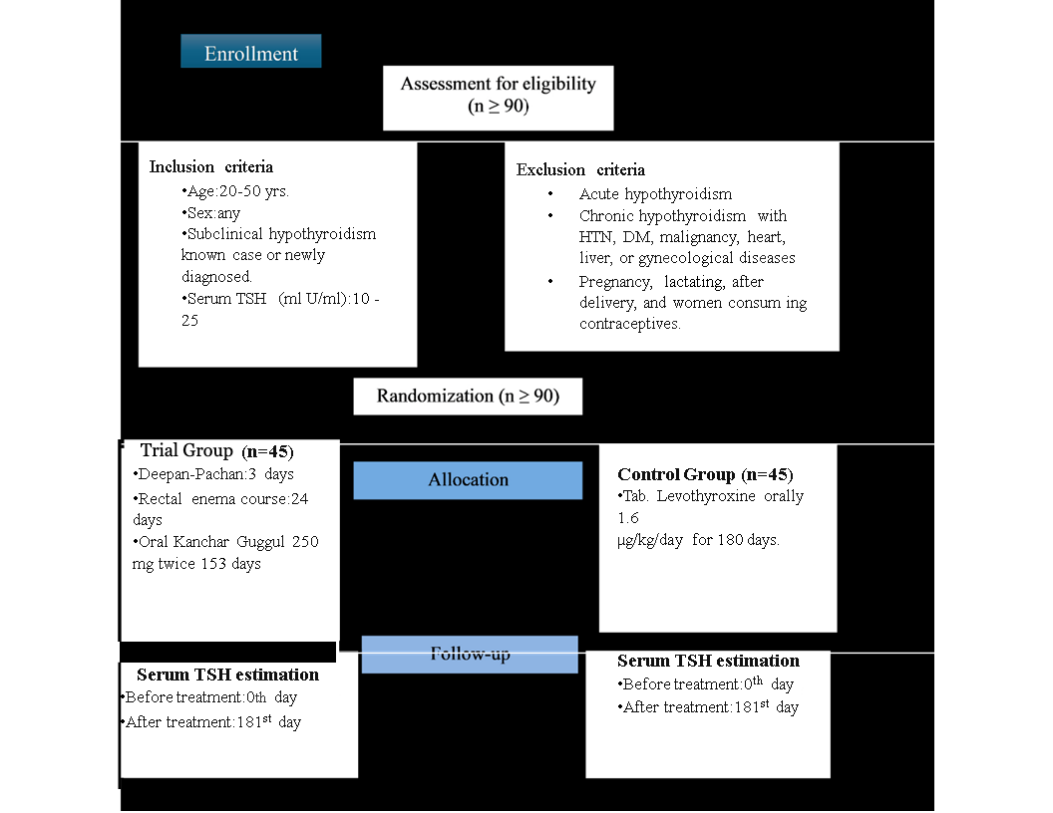
Study design. DM: diabetes mellitus; HTN: hypertension; TSH: thyroid stimulating hormone.

This is a single-blind, RCT that aims to determine the superiority of one treatment over another (Figure 1). The study consists of 2 arms and follows a prospective design. Participants will be randomly assigned to either arm in a 1:1 ratio.

**Figure 2:**
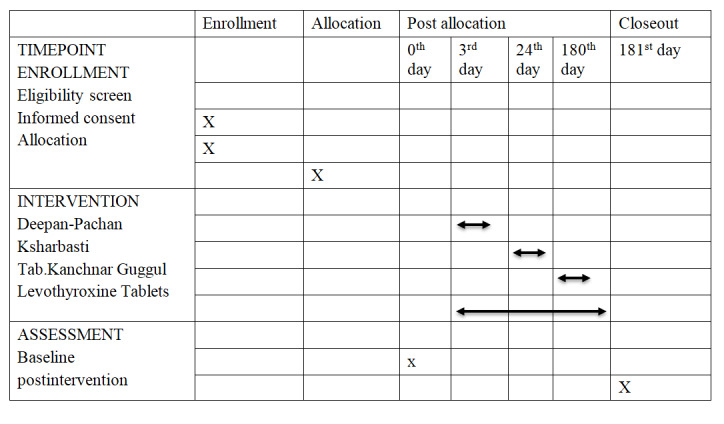
SPIRIT (Standard Protocol Items: Recommendations for Interventional Trials) flowchart.

The study will be carried out in the Panchakarma department of the hospital affiliated with Mahatma Gandhi Ayurvedic College, hospital, and Research Center (Salod, Wardha district, Maharashtra, India). This study will be conducted following SPIRIT standards for RCTs (Figure 2).

### Diagnostic Criteria

The diagnosis of SCH will be made per the *ICD-10* (*International Statistical Classification of Diseases, Tenth Revision*) criteria (SCH) as follows: TSH>4.50 µIU/mL, fT4=0.8-1.8 ng/dL, fT3=1.4-4.4 Pg/mL [13] (Textbox 1).

**Eligibility, exclusion, withdrawal, and adverse drug reaction criteria.**

**Eligibility criteria:**

1. Age: 20-60 years
2. Sex: Any
3. Known case, or newly diagnosed case of subclinical hypothyroidism

**Exclusion criteria:**

1. Overt hypothyroidism
2. Subclinical hypothyroidism with Diabetic Mellitus, hypertension, pregnancy, and gynecological disorders
3. Hashimoto thyroiditis
4. Kshar Basti contra-indications

**Withdrawal criteria:**

The participant may be withdrawn from the trial if there is one of the following:

1. Any adverse event and adverse drug reaction (ADR) occurrence
2. Voluntary withdrawal by the participant
3. Violation of inclusion and exclusion criteria
4. There is noncompliance with the treatment regimen (a minimum of 80% compliance is essential to continue in the study)

**ADR**

If an ADR occurs during the trial, the participant will be treated at the institute-attached hospital, and the investigators will cover the costs.

**Criteria for discontinuing from the trial**

If the participant breaks the sequence of panchakarma therapy and misses the oral therapy for ≥7 days, he/she will not be considered in the trial.

### Grouping

The grouping of participants is done in a parallel design where Group A (the trial group) consists of 45 participants and Group B (the control group) consists of 45 participants.

### Interventions

In this randomized clinical trial, Group A, referred to as the trial group, will be administered conventional treatment, while Group B, referred to as the control group, will be administered Ayurvedic treatment (Textbox 2 and Figure 1).

**Interventions.**

- For Group A (the trial group), the therapeutic decoction enema (Kshar Basti) with oil massage to the whole body, followed by hot fomentation before the enema, will be administered for 8 days. We will use the Course of Eight Therapeutic Enemas (Yoga Basti) method. We will use plain sesame oil for the therapeutic unctuous enema (Anuvasan Basti).
- For Group B (the control group), levothyroxine tablets at a dose of 1.6 µg/kg/day will be administered for 180 days.

### Randomization

We will apply random allocation to recruit the participants in the study. Computer-generated random numbers will be used.

### Blinding

The allocation will be researcher blind. A third person, such as a nurse or panchakarma therapist, will help to allocate the participant using computer-generated random numbers. Since this study is researcher-blind, a PhD guide will allocate and assign the participants.

### Screening Investigations

Serum TSH will be measured before and after the study. On the 181st day, serum TSH, T_3_, and T_4_ will be measured. The above findings will be compared to the baseline findings.

### Criteria of the Assessment or Outcome Variable

A variation of more than 20% of the baseline value of serum TSH with normal values of T_3_ and T_4_ will be considered to determine the efficacy of the intervention. Therefore, decreasing serum TSH with no change in T_3_ and T_4_ means, Kshar Basti with Kanchanar Guggul is efficacious in SCH.

### Interventions for the Study Group

#### Deepan-Pachan

Shivakshar Pachak Churna is an Ayurvedic medication that should be taken orally, with a dosage of 1 gram, twice a day, for 3 days (Table 1). Before commencing Basti therapy, it will enhance the appetite, a crucial factor. The preparation will be conducted in the pharmacy that is connected to our institute.

**Table 1:** Contents of Shivakshar Pachak Churna.

Ayurvedic name	Latin name/English name
Sunthi	*Zingiber officinale*
Marich	*Piper nigrum*
Pippali	*Piper longum*
Ajmoda	*Trachyspermun roxburghianum*
Jeeraka	*Cuminum cyminum*
Krishnajeerak	*Nigella sativa*
Hingu	*Ferula asafoetida*
Haritaki	*Terminalia chebula*
Saindhav	Rock salt
Sajjikshar	Carbonate of soda

#### Kanchanar Guggul [10]

It will be prepared in the pharmacy attached to our hospital. It will be designed per the standard operating procedure (SOP) mentioned in the pharmacy attached to the hospital. The contents are provided in the table (Table 2).

**Table 2:** Contents of Kanchnar Guggul.

Ayurvedic name	Latin name
Kanchanar	*Bauhinia variegata linn*
Amalaki	*Embelica officinalis*
Haritaki	*Terminalia chebula*
Bhibtak	*Terminalia bellirica*
Pippali	*Piper longum*
Varuna	*Crateva religiosa*
Dalchini	*Cinnamomum cassia*
Ela	*Elettaria cardamomum*
Tejpatra	*Cinnamomum tamala*
Guggul	*Commiphora wightii*

#### Kshar Basti and Its Procedure

##### Preoperative

**Figure 3:**
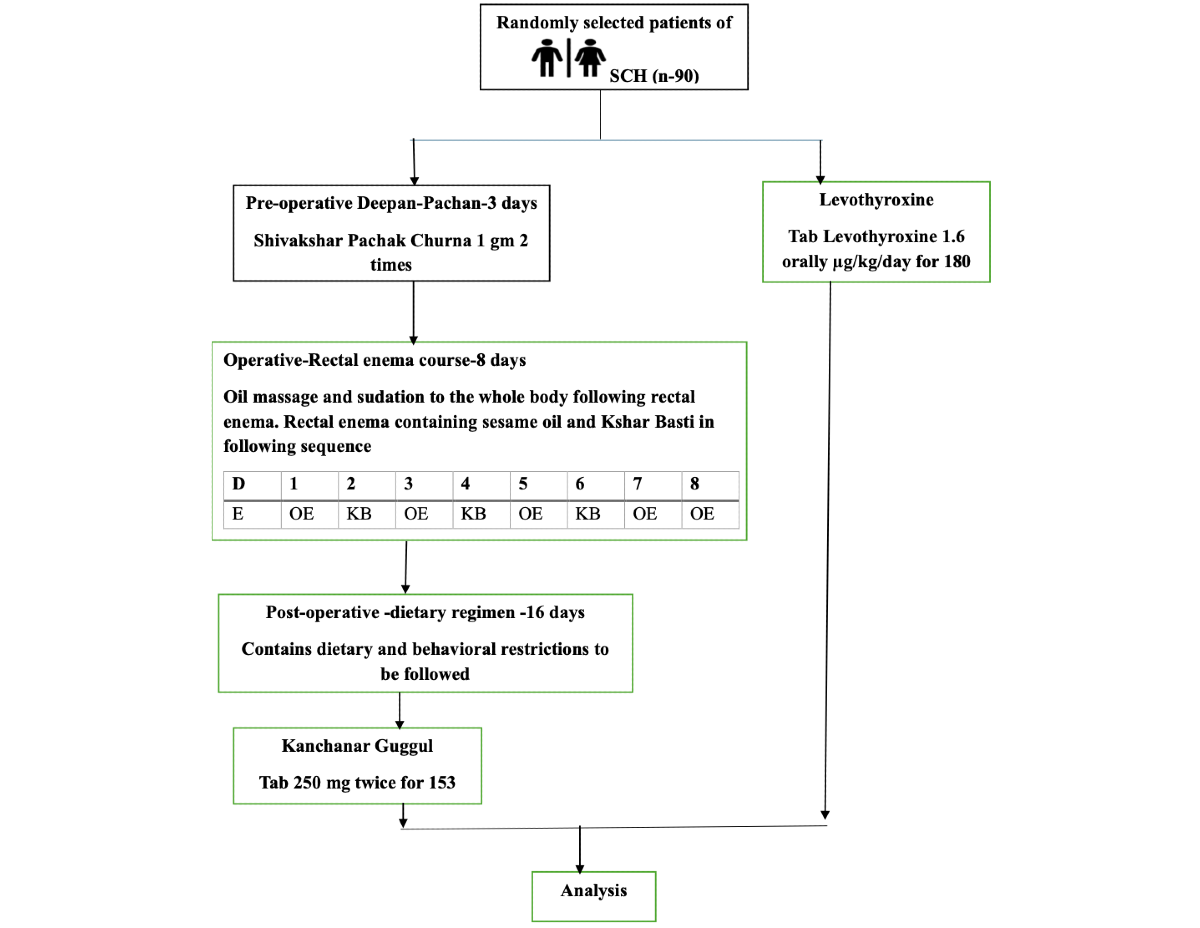
Intervention details. KB: Kshar Basti; OE: oil enema; SCH: subclinical hypothyroidism.

Kshar Basti [12] is a therapeutic decoction enema [14]. Therefore, we will follow the same operative procedures that apply to Niruha Basti. The Yoga Basti method (Figure 3) will administer Niruh Basti and Anuvasan Basti (therapeutic unctuous enemas) on alternate days, except the last day. For 3 days, we will administer Deepan-Pachan, which enhances metabolic fire. Kshar Basti’s decoction will be prepared per the SOPs followed in the hospital. Table 3 lists Kshar Basti's contents. For the Anuvasan Basti, we will use plain sesame oil. We will call the participants to the hospital early for the Kshar Basti during the day (before 3 PM). Sesame oil massage and hot fomentation will be performed on the whole body. The fomentation will be conducted as per the SOPs of the hospital as mentioned by the National Accreditation Board for Hospitals.

**Table 3:** Contents of Kshar Basti.

Content	Quantity
Jaggery (Guda)	100 g
Tamarind pulp (Amlika)	100 g
Rock salt (Saindhav)	10 g
Cow’s urine (Gomutra)	400 mL
Paste of Anthem Sova (Shatavha Kalka)	10 g
Total	500 mL

##### Operative

The raw medicines will be collected, prepared, and made lukewarm by indirect heating. Materials will be taken into an enema can. The participant will be asked to lie over the table in the left lateral position. The nozzle of the enema can be slowly inserted into the anal canal after applying oil to it. It will be slowly inserted till it reaches the rectum, and now the decoctions will enter the rectum. Soon after the administration of Kshar Basti, the participant will be allowed to defecate.

##### Postoperative Period

The participant will be asked to follow a dietary regimen like Yusha (a soup of vegetables or pulses) and rice and drink hot water during the Kshar Basti course.

##### Course Duration

Basti will be administered daily for 8 days; after that, the participant must follow the regimen for 16 days. The total duration of the course will be 27 days (including Deepan-Pachan for 3 days), and after this course, Kanchanar Guggul will be administered for 153 days. The duration of the therapy will be 180 days, and the follow-up period will be 180 days.

Both groups of patients are restricted from taking or consuming any medications, devices, or nonpharmacological treatments that have an impact on hypothyroidism.

### Sample Size Calculations

We used the following formula for the determination of sample size (N=40) [15]:

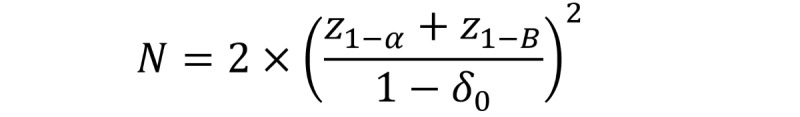



Thus, there will be 45 participants in each group (5 added to minimize the error), and our study’s sample size will be 90 (n=90).

We also noted the sample size utilized in past studies conducted on hypothyroidism at this institute. Thus, a sample size of 90 would be both practical and adequate.

(N=size per group; *z*_1–_*_α_*= the standard normal deviate for one group; *z*_1–_*_β_*= the standard normal deviate for the other group, δ_0_= a clinically acceptable margin.)

### Data Collection Methods

The researcher will gather and archive the data. The data will be collected using appropriate software, such as Epi-Info. We will examine the data after the trial concludes. Replicates will be discarded. We will choose a conventional laboratory to quantify serum TSH.

### Data Confidentiality

Only the PhD guide will have access to the acquired data, which includes biological samples from the individuals. However, upon completion of the study, any researcher may request access to the data for a systematic review and meta-analysis.

### Audit

We will conduct the audit 6 months after the trial begins, under the supervision of a PhD mentor. Furthermore, we will closely observe and track any negative reactions caused by the drugs or treatments used in our operations, as well as ensure strict adherence to the established protocol.

We will educate the participants about the favorable attributes of Kshar Basti (the therapeutic enema), to ensure their compliance with the treatment regimen and adherence to the established procedures. We will closely observe the reduction in participants from this experiment and the factors contributing to this reduction.

### Statistical Analysis

After summarizing the data, we will use the Kolmogorov-Smirnov test to decide if the data are following a normal distribution. If normally distributed, the student *t* test will be used; otherwise, the z-test will be applied. We will use IBM SPSS (version 26) for statistical analysis. We will set the *P* value to less than .05 for statistical significance.

## Results

### Overview

We will examine the following factors in the demographics: age, gender, location, smoking behavior, alcohol consumption, and the type of salt used to achieve the secondary purpose of this study. We will examine the rate at which participants drop out of the Kshar Basti course, as well as any occurrences of difficulties associated with the Kshar Basti course.

We will only recruit participants for this study once. We will finish the drug preparation and acquisition by June 2024. The projected timeline for the recruitment process is June 2024 to December 2025. We will evaluate the information right away and conclude at the start of 2026.

### Evaluation Outcomes

The primary assessment result of our study is the disparity in the TSH, T3, and T4 levels compared to the initial values. We will deem any deviation of 20% or more from the baseline as statistically significant.

## Discussion

### Principal Findings

The 45 participants in the control group will receive oral L-thyroxine. On the other hand, the trial group, also consisting of 45 participants, will get a treatment involving Kshar Basti and oral Kanchnar Guggul tablets. Both groups will undergo treatment for 180 days, and on day 181, we will assess the levels of TSH, T3, and T4 in the blood. We will compare the changes before and after therapy. We expect that the Ayurvedic intervention, specifically the combination of Kshar Basti and Kanchnar Guggul, will demonstrate statistically significant outcomes compared to the standard treatment. This would establish Ayurvedic intervention as a viable treatment option for SCH, eliminating the need for lifelong consumption of L-thyroxine tablets by the patient.

Hashimoto thyroiditis typically correlates with hypothyroidism. The thyroid gland produces numerous growth and vasoactive substances. The epithelial cells of the thyroid gland contain the glycoprotein known as vascular endothelial growth factor. It plays a critical role in the elevated levels of TSH in SCH.

A study compared the administration of Nigella sativa powder at a dosage of 2 grams per day for 8 weeks with the administration of placebos consisting of starches at the same dosage and duration. This RCT involved 47 human participants diagnosed with Hashimoto thyroiditis. Nigella sativa treated 23 of them, while starches served as a placebo for the remaining 24. The study determined that Nigella sativa seeds were useful in alleviating Hashimoto thyroiditis. It also successfully reduced vascular endothelial growth factor levels and body weight.

In Ayurveda, Nigella sativa is referred to as “Krishnajeeraka.” In our study, we will be administering Shivakshar Pachak Churna as “Deepan-Pachan” for the first 3 days. Nigella sativa is a constituent of Shivakshar Pachak Churna [16].

In a separate trial [17], the authors randomly assigned 53 individuals with hypothyroidism to either the treatment group or the control group, administering a placebo to the latter. The ginger group, consisting of 27 participants, was given a 500 mg capsule containing ginger twice a day, whereas the placebo group, consisting of 26 participants, was given starch. This treatment lasted for 30 days. Both groups were administered L-thyroxine. The thyroid symptoms rating questionnaire was used to evaluate the results. The initial findings of this trial showed that individuals with primary hypothyroidism who consumed ginger powder daily while receiving appropriate hormone replacement therapy (achieving biochemical euthyroid) experienced a notable decrease in symptoms associated with hypothyroidism. Our study incorporates Zanzibar as a component of the “Shivaksharpachak Churna.”

Basti therapy is recommended for the treatment of numerous physical and psychological conditions [18]. Immunomodulation was observed following Basti, according to a study that evaluated the metabolic and immunologic response to Basti therapy. One potential mechanism by which it operates is by modulating T cells, immunoglobins, and proinflammatory mediators.

A study [17] found that over 50% of people with hypothyroidism have small intestinal bacterial overgrowth (SIBO). The study found that 54% of hypothyroidism patients tested positive for SIBO using the glucose breath test, compared to 5% in the control group. Diarrhea is a common symptom among hypothyroidism patients, with a positive hydrogen breath test and antibiotic response indicating SIBO. Hypothyroidism can cause less movement in the gastrointestinal tract, leading to long-lasting gastrointestinal problems.

An additional investigation [18] demonstrated that 300 mg of Ashwagandha root extract twice daily for 8 weeks produced statistically significant outcomes. The impact of Ashwagandha root extract on the hypothalamic-pituitary-thyroid axis was hypothesized by the authors. Ashwagandha has antidopaminergic and anti-inflammatory properties. These characteristics may contribute to the thyroid-modulating effect.

Kshar Basti is a therapeutic mixture containing Tamarindus pulp, which acts as a laxative and helps alleviate gastrointestinal troubles [19]. Jaggery is beneficial for the gut microbiota [20]. It is believed that Shatavha (Anthem sowa) possesses estrogenic characteristics [21]. It affects the blood, bones, and central nervous system. The rock salt contains sodium, potassium, and chloride ions [22]. It causes the solution to become hypertonic. Guggul is derived from the latex of the Guggul plant. Recently, an article revealed the antihypothyroidism properties of the Guggul herb [23]. Therefore, the Ayurvedic treatment involving Kshar Basti and Kanchnar Guggul is believed to enhance the immune system and alleviate SCH.

The efficacy of Kshar Basti and Kanchanar Guggul in the treatment of hypothyroidism can be substantiated with dependable evidence in this study, owing to its RCT design and moderate sample size of 90. In addition, the duration of the study will be 180 days, which is sufficient to detect fluctuations in TSH and thyroid hormone levels.

### Principal Results

The primary findings will establish that the Ayurvedic therapy including Kshar Basti (administration of herbal decoction through enema) followed by oral administration of Tab Kanchanar Guggul is effective in treating hypothyroidism.

### Publication Plan

We intend to disseminate the results of our study by publishing it as either an original research article or a short communication in a journal that is indexed.

### Limitations

Our investigation has some limitations. To begin with, 90 samples is still a moderate amount. To account for this prevalence rate, a minimum of 300 samples would be adequate. The duration of treatment is extended. Consequently, a large dropout rate is possible. Hence, a practical sample size of 90 was selected.

The preventive effect of these Ayurvedic interventions on SCH cannot be determined through our research. To accomplish this, an additional observational study with a minimum 1-year follow-up period is required.

### Conclusions

Our proposed study will evaluate the effectiveness of Ayurvedic intervention, specifically Kshar Basti and oral Kanchanar Guggul tablets, in managing SCH. Additionally, we will assess the appropriateness of the Kshar Basti course. The findings of this study will provide evidence for the use of Ayurvedic treatment as an option for SCH.

We suggest doing a further investigation to assess the independent effectiveness of Kshar Basti and Kanchanar Guggul in alleviating OH. There is a need for a study to assess the effectiveness of these Ayurvedic therapies in treating hypothyroidism during pregnancy.

## Appendix

Multimedia Appendix 1 SPIRIT checklist.

## Abbreviations

*ICD-10*: *International Statistical Classification of Diseases, Tenth Revision*
IEC: Institutional Ethical Committee
L-thyroxine: levothyroxine
OH: overt hypothyroidism
RCT: randomized controlled trial
SCH: subclinical hypothyroidism
SIBO: small intestinal bacterial overgrowth
SOP: standard operating procedure
T3: triiodothyronine
T4: thyroxine
TRUST: Thyroid Hormone Replacement for Untreated Older Adults with Subclinical Hypothyroidism Trial
TSH: thyroid stimulating hormone
