# Supplementary material for: Evaluation of Comparative Efficacy of Levothyroxine Versus Kshar Basti and Kanchanar Guggul in the Treatment of Hypothyroidism: Protocol for a Randomized Controlled Trial
Source: JMIR Res Protoc. 2024 Sep 10;13:e57287. doi: 10.2196/57287 (PMC11422726; doi:10.2196/57287)
Supplement: Multimedia Appendix 1 [file resprot_v13i1e57287_app1.pdf]

Section/item	Item No	Description	Addressed on page number
**Administrative information**			
Title	1	Descriptive title identifying the study design, population, interventions, and, if applicable, trial acronym	Page 1, lines 1-3.
Trial registration	2a	Trial identifier and registry name. If not yet registered, name of intended registry	Page 1, lines 28-29
	2b	All items from the World Health Organization Trial Registration Data Set	Page 1, Lines 28 - 29
Protocol version	3	Date and version identifier	Page 1, lines 28 - 19
Funding	4	Sources and types of financial, material, and other support	Page 14, line 453 - 454
Roles and responsibilities	5a	Names, affiliations, and roles of protocol contributors	Title page
	5b	Name and contact information for the trial sponsor	This trial is not sponsored.
	5c	Role of study sponsor and funders, if any, in study design; collection, management, analysis, and interpretation of data; writing of the report; and the decision to submit the information for publication, including whether they will have ultimate authority over any of these activities	NA
	5d	Composition, roles, and responsibilities of the coordinating centre, steering committee, endpoint adjudication committee, data management team, and other individuals or groups overseeing the trial, if applicable (see Item 21a for data monitoring committee)	NA
**Introduction**			,

Background and rationale	6a	Description of research question and justification for undertaking the trial, including summary of relevant studies (published and unpublished) examining benefits and harms for each intervention	Page 2, lines 64 - 82.
	6b	Explanation for choice of comparators	Page 2, lines 75 - 82.
Objectives	7	Specific objectives or hypotheses	Page 3, lines 103-114.
Trial design	8	Description of trial design including type of trial (e.g., parallel-group, crossover, factorial, single group), allocation ratio, and framework (e.g., superiority, equivalence, noninferiority, exploratory)	3 Page -4, lines 132-161
**Methods: Participants, interventions, and outcomes**			
Study setting	9	Description of study settings (eg, community clinic, academic hospital) and list of countries where data will be collected. Reference to where list of study sites can be obtained	Page 4. lines 132-140
Eligibility criteria	10	Inclusion and exclusion criteria for participants. If applicable, eligibility criteria for study centers and individuals who will perform the interventions (e.g., surgeons, psychotherapists)	Textbox 2 Page 9
Intervention s	11 a	Interventions for each group with sufficient detail to allow replication, including how and when they will be administered	4 Page, lines 150 - 153, page 9 textbox 2
	11 b	Criteria for discontinuing or modifying allocated interventions for a given trial participant (eg, drug dose change in response to harms, participant re quest, or improving/worsening disease)	Page 9, Textbox 2, line 272
	11c	Strategies to improve adherence to intervention protocols, and any procedures for monitoring adherence (eg, drug tablet return, laboratory tests)	Page 6 -7, lines 251, 252
	11 d	Relevant concomitant care and interventions that are permitted or prohibited during the trial	Page 6, lines 217, 218

Outcomes	12	Primary, secondary, and other outcomes, including the specific measurement variable (eg, systolic blood pressure), analysis metric (eg, change from baseline, final value, time to event), method of aggregation (eg, median, proportion), and time point for each outcome. Explanation of the clinical relevance of chosen efficacy and harm outcomes is strongly recommended	Page 6, lines 266-269
Participant timeline	13	Schedule of enrolment, interventions (including any run-ins and washouts), assessments, and visits for participants. A schematic diagram is highly recommended (see Figure)	Page 8, Textbox 1 study design, line 271
Sample size	14	Estimated number of participants needed to achieve study objectives and how it was determined, including clinical and statistical assumptions supporting any sample size calculations	Page 6-7 lines 250-253.
Recruitment	15	Strategies for achieving adequate participant enrolment to reach target sample size	Page 6-7, line 250 - 253
**Methods: Assignment of interventions (for controlled trials)**			
Allocation:			
Sequence generation	16a	Method of generating the allocation sequence (eg, computer-generated random numbers), and list any factors for stratification. To reduce predictability of a random sequence, details of any planned restriction (eg, blocking) should be provided in a separate document that is unavailable to those who enrol participants or assign interventions	Page 4, Lines 154-156.
Allocation concealment mechanism	16b	Mechanism of implementing the allocation sequence (eg, central telephone; sequentially numbered, opaque, sealed envelopes), describing any steps to conceal the sequence until interventions are assigned	Page 4, lines -157-161
Implementation	16c	Who will generate the allocation sequence, who will enrol participants, and who will assign participants to interventions	Page 4, Lines 157 - 161.

Blinding (masking)	17 a	Who will be blinded after assignment to interventions (eg, trial participants, care providers, outcome assessors, data analysts), and how	Page 4, lines 157-161.
	17 b	If blinded, circumstances under which unblinding is permissible, and procedure for revealing a participant's allocated intervention during the trial	NA
**Methods: Data collection, management, and analysis**			
Data collection methods	18 a	Plans for assessment and collection of the outcome, baseline, and other trial data, including any related processes to promote data quality (eg, duplicate measurements, training of assessors) and a description of study instruments (eg, questionnaires, laboratory tests) along with their reliability and validity, if known. Reference to where data collection forms can be found, if not in the protocol	Page 6, lines 234-238
	18 b	Plans to promote participant retention and complete follow-up, including list of any outcome data to be collected for participants who discontinue or deviate from intervention protocols	Page 6-7, lines 250 – 253
Data managemen t	19	Plans for data entry, coding, security, and storage, including any related processes to promote data quality (eg, double data entry; range checks for data values). Reference to where details of data management procedures can be found, if not in the protocol	Page 6, lines 234-238.
Statistical methods	20 a	Statistical methods for analysing primary and secondary outcomes. Reference to where other details of the statistical analysis plan can be found, if not in the protocol	Page 7 lines -254-259.
	20 b	Methods for any additional analyses (eg, subgroup and adjusted analyses)	NA
	20c	Definition of analysis population relating to protocol non-adherence (eg, as randomised analysis), and any statistical methods to handle missing data (eg, multiple imputation)	Page 6 -7 lines 250 -253.
**Methods: Monitoring**			
Data monitoring	21 a	Composition of data monitoring committee (DMC); summary of its role and reporting structure; statement of whether it is independent from the sponsor and competing interests; and reference to where further details about its charter can be found, if not in the protocol. Alternatively, an explanation of why a DMC is not needed	Page 6, line 234 -238

	21 b	Description of any interim analyses and stopping guidelines, including who will have access to these interim results and make the final decision to terminate the trial	NA
Harms	22	Plans for collecting, assessing, reporting, and managing solicited and spontaneously reported adverse events and other unintended effects of trial interventions or trial conduct	Page 8-9, inside textbook 2, line 272
Auditing	23	Frequency and procedures for auditing trial conduct, if any, and whether the process will be independent from investigators and the sponsor	Page 6, line 245 -249.
**Ethics and dissemination**			
Research ethics approval	24	Plans for seeking research ethics committee/institutional review board (REC/IRB) approval	Page 3, lines 123-126.
Protocol amendments	25	Plans for communicating important protocol modifications (eg, changes to eligibility criteria, outcomes, analyses) to relevant parties (eg, investigators, REC/IRBs, trial participants, trial registries, journals, regulators)	NA
Consent or assent	26 a	Who will obtain informed consent or assent from potential trial participants or authorised surrogates, and how (see Item 32)	Page 3, lines -127 - 128.
	26 b	Additional consent provisions for collection and use of participant data and biological specimens in ancillary studies, if applicable	Page 3, lines 130-131
Confidentiality	27	How personal information about potential and enrolled participants will be collected, shared, and maintained to protect confidentiality before, during, and after the trial	Page 6, lines -241-244.
Declaration of interests	28	Financial and other competing interests for principal investigators for the overall trial and each study site	Page 14, lines 451-452.
Access to data	29	Statement of who will have access to the final trial dataset, and disclosure of contractual agreements that limit such access for investigators	Page 6, line 241 -244.

Ancillary and post-trial care	30	Provisions, if any, for ancillary and post-trial care, and for compensation to those who suffer harm from trial participation	Page 9-9 Textbox 2, line 272
Disseminati on policy	31 a	Plans for investigators and sponsor to communicate trial results to participants, healthcare professionals, the public, and other relevant groups (eg, via publication, reporting in results databases, or other data-sharing arrangements), including any publication restrictions	Page 14, lines 423-424
	31 b	Authorship eligibility guidelines and any intended use of professional writers	NA
	31c	Plans, if any, for granting public access to the full protocol, participant-level dataset, and statistical code	NA
**Appendices**			
Informed consent materials	32	Model consent form and other related documentation given to participants and authorised surrogates	Attached
Biological specimens	33	Plans for collection, laboratory evaluation, and storage of biological specimens for genetic or molecular analysis in the current trial and future use in ancillary studies, if applicable	NA
